# Automated Label-Free Classification of Circulating Tumor Cells and White Blood Cells Using Hyperspectral Imaging and Deep Learning on Microfluidic SACA Chip System

**DOI:** 10.3390/mi17040472

**Published:** 2026-04-14

**Authors:** Shun-Chi Wu, Jon-Nan Chiu, Yi-Wen Chen, Chen-Hsi Hung, Mang Ou-Yang, Fan-Gang Tseng

**Affiliations:** 1 Department of Engineering and System Science, National Tsing Hua University, Hsinchu 30013, Taiwan; shunchi.wu@mx.nthu.edu.tw (S.-C.W.); s111011530@m111.nthu.edu.tw (J.-N.C.); yiwenchen890516@gmail.com (Y.-W.C.); alex0979070936@gmail.com (C.-H.H.); 2 Department of Electronics and Electrical Engineering, National Yang Ming Chiao Tung University, Hsinchu 11217, Taiwan; oym.mail@gmail.com; 3 Research Center for Applied Sciences, Academia Sinica, Taipei 115201, Taiwan

**Keywords:** circulating tumor cells, hyperspectral imaging, deep learning, label-free, ResNet, ensemble voting, microfluidics

## Abstract

Circulating tumor cells (CTCs) are essential biomarkers for cancer prognosis, yet their extreme rarity and biological heterogeneity pose significant challenges for label-free detection. This study presents an automated, non-invasive classification framework integrating a self-assembly cell array (SACA) microfluidic chip with hyperspectral imaging (HSI) and deep learning. By utilizing the SACA chip’s 5 µm gap design, patient-derived blood samples were organized into a flattened monolayer, ensuring high-purity spectral acquisition by minimizing cell overlapping. We implemented two deep-learning pipelines: an Attention-Based Adaptive Spectral–Spatial Kernel ResNet (A^2^S^2^K-ResNet) for pixel-level feature extraction and a modified ResNet50 for structural image analysis. While spectral classification achieved ~80% accuracy for cultured cell lines, its performance on patient-derived CTCs was hindered by subtle spectral overlap with white blood cells (WBCs). To overcome this, a multi-band ensemble strategy using majority voting across seven optimized spectral bands (470–900 nm) was developed. This hybrid approach significantly enhanced detection robustness, achieving an overall accuracy of >93.5% and precision exceeding 92%. These results demonstrate that combining microfluidic spatial control with multi-band deep learning offers a reliable, label-free pipeline for clinical liquid biopsy and real-time cancer monitoring.

## 1. Introduction

Circulating tumor cells (CTCs) are cancer cells that detach from primary solid tumors, enter the bloodstream, and migrate to distant sites, where they may initiate metastatic growth [1]. As key biomarkers for understanding tumor biology and disease progression, CTCs provide useful information for early cancer diagnosis, as well as treatment monitoring and prognosis. Their detection enables timely clinical intervention before significant disease advancement, potentially improving patient outcomes [2]. Moreover, fluctuations in CTC counts serve as valuable indicators of therapeutic response, guiding clinicians in adapting treatment strategies accordingly [3,4,5,6,7]. Importantly, CTC detection provides a non-invasive alternative for cancer monitoring, particularly beneficial for patients who cannot undergo repeated invasive procedures.

CTC enumeration has been recognized as an independent prognostic factor in several malignancies, including breast and prostate cancer, with CTC counts closely correlating with treatment outcomes [3,4,5]. Similar prognostic significance has been observed in lung cancer patients [7]. These findings underscore the clinical utility of tracking CTC dynamics to evaluate disease progression and optimize therapeutic regimens.

However, a major challenge in CTC detection lies in their extreme rarity. Typically, only 1–10 CTCs are found in every 10 mL of peripheral blood [8], amidst an overwhelming background of approximately 4.5–10 × 10^6^ white blood cells and 3.5–5.9 × 10^9^ red blood cells per milliliter [9]. Detecting such rare cells among the vast numbers of normal blood cells remains technically challenging and demands highly sensitive and specific detection technologies. Complicating matters further, CTCs exhibit pronounced biological and biochemical heterogeneity. For instance, median diameters of CTCs vary substantially by cancer type: 12.4 µm in breast cancer, 10.3 µm in prostate cancer, 7.5 µm in colorectal cancer, and 8.6 µm in bladder cancer. Significant intra-tumor variability in CTC size has also been reported [10].

In terms of molecular phenotype, expression of the epithelial cell adhesion molecule (EpCAM)—a widely used CTC biomarker—shows considerable variability across cancer types. Immunohistochemical analysis using the Total Immunostaining Score (TIS) has revealed heterogeneous EpCAM expression among colorectal, gastric, pancreatic, gallbladder, and hepatocellular cancers, ranging from low (TIS 1–4) to moderate (TIS 6–8) and high (TIS 9–12) levels [11]. This variability limits the effectiveness of universal biomarker-based isolation approaches.

Fluorescence-based CTC detection methods, while precise, face several limitations. These include dependence on antibody-labeled probes targeting protein overexpression—which may vary by tumor type and individual patient [12]—as well as issues such as photobleaching, phototoxicity, and the requirement accordingly for expert dye selection [13]. Furthermore, fluorescent labeling can compromise cell viability, restricting subsequent analyses such as cell culture or functional testing [14,15].

As an alternative, label-free techniques such as hyperspectral imaging (HSI) allow for rapid, non-invasive cell analysis, capturing spectral and morphological data without the need for immunofluorescence or labels. Unlike conventional staining methods, HSI preserves cell viability and eliminates the need for exogenous labels. In recent years, HSI has gained increasing attention in life sciences and biomedical imaging, offering complementary capabilities alongside quantitative phase imaging (QPI) and Raman spectroscopy (RS) [16]. Various HSI modalities—including reflectance hyperspectral microscopy, hyperspectral Raman imaging [17,18], and multispectral fluorescence imaging [19]—have demonstrated potential for both stained and label-free biological applications [20]. For example, Bertani et al. successfully distinguished melanoma cells from keratinocytes using hyperspectral confocal reflectance microscopy [21] and further applied the same technique to semi-automatically classify macrophage polarization states [22].

Recent developments in deep learning have enabled more efficient processing of hyperspectral image data, especially for complex biomedical imaging tasks.Deep learning architectures provide powerful frameworks for representation learning, allowing for the automatic discovery of intricate structures in high-dimensional data [23]. Since the introduction of AlexNet [24], convolutional neural networks (CNNs) have achieved significant breakthroughs across a wide range of image recognition tasks [25]. Among them, deep residual networks (ResNets), which utilize two-dimensional CNN architectures, have shown exceptional capability in extracting hierarchical spatial features from images [26]. In parallel, one-dimensional CNNs [27] and recurrent neural networks such as long short-term memory (LSTM) models [28] have been increasingly applied to spectral sequence data, demonstrating strong performance in clustering and classifying time-series signals [29].

Recent advancements have positioned the intersection of microfluidics and artificial intelligence (AI) as a transformative frontier in biomedical engineering. Beyond cell classification, machine learning (ML) tools are increasingly leveraged to optimize complex microfluidic processes across diverse chip architectures. For instance, deep learning models have been successfully applied to predict and control droplet formation dynamics in real-time [30]. Furthermore, reinforcement learning algorithms are now utilized to automate the design optimization of microchannel geometries, significantly reducing the trial-and-error cycle in device fabrication [31]. In the context of bioanalysis, AI-driven platforms enable high-throughput screening of rare biomarkers by integrating automated flow control with rapid image processing [32,33]. These intelligent systems also facilitate the optimization of mixing efficiency in passive micromixers and the precise sorting of heterogeneous cell populations [34,35]. Moreover, the application of neural networks in analyzing impedance and acoustic signals within microchips has enhanced the sensitivity of label-free diagnostic tools [36,37]. By leveraging these ML-based strategies, researchers can achieve superior stability and reproducibility in microfluidic-based liquid biopsies [38,39].

**Figure 1:**
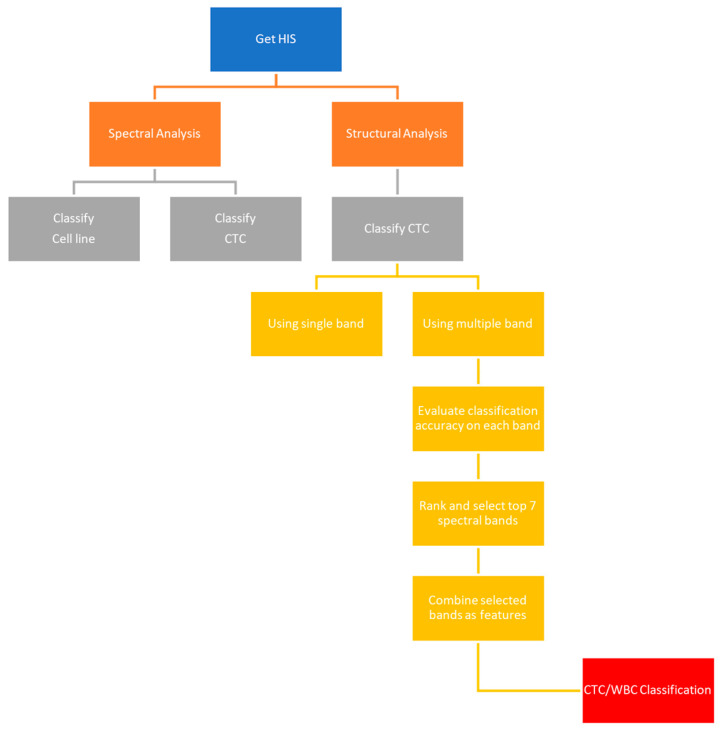
Overview of the cell classification procedure used in the materials and methods of this research.

Based on these prior findings, we developed an approach that combines hyperspectral imaging with deep learning models to identify circulating tumor cells (CTCs) in blood samples without the use of labels. While current CTC enrichment methods largely depend on immunofluorescent labeling—which, despite its high specificity, is labor-intensive and compromises cell viability—our method seeks to establish an automated, label-free detection pipeline. The overall workflow is illustrated in Figure 1. Although hyperspectral information alone is often insufficient to reliably distinguish CTCs from white blood cells (WBCs), we address this limitation by incorporating structural analysis and a multi-band voting strategy. This combined strategy improves classification performance by incorporating deep learning models. We expect that this platform could support non-invasive cancer diagnostics and assist in real-time clinical monitoring.

## 2. Methods

This study aimed to develop an automated, label-free classification framework capable of distinguishing circulating tumor cells (CTCs) from white blood cells (WBCs) using hyperspectral imaging (HSI) and deep learning. The goal was to establish a rapid and non-invasive pipeline that could potentially support early cancer detection and clinical monitoring. The experimental work was jointly conducted by the Biomedical Engineering and Microfluidic Systems Laboratory, Department of Engineering and System Science, National Tsing Hua University, and the Department of Electrical and Computer Engineering, National Yang Ming Chiao Tung University, Hsinchu, Taiwan. Peripheral blood samples obtained from colorectal cancer patients were processed and analyzed using a customized HSI system to capture both spectral and structural cellular information. Two deep learning models were implemented: A^2^S^2^K-ResNet for spectral–spatial feature extraction and a modified ResNet50 for structural image classification. Model performance was assessed using five-fold cross-validation with accuracy, precision, recall, and kappa statistics as evaluation metrics. No formal power calculation was performed, as the study focused on developing and validating a computational framework rather than testing a clinical hypothesis.

### 2.1. Sample Preparation

This study was conducted in collaboration with Taipei Veterans General Hospital and National Yang Ming Chiao Tung University, as illustrated in Supplementary Figure S8, with the objective of detecting circulating tumor cells (CTCs) in colorectal cancer patients and analyzing epithelial–mesenchymal transition (EMT) characteristics using hyperspectral imaging. Peripheral blood samples were collected in cell preparation tubes (BD Vacutainer^TM^ CPT tubes, BD Biosciences, Franklin Lakes, NJ, USA), which contain Ficoll Hypaque, anticoagulants, and a polyester gel. Upon centrifugation, the gel forms a barrier separating mononuclear cells from other blood components, allowing for stable sample storage and downstream processing.

#### 2.1.1. Reagents and Staining Protocol

Buffer and Media:

Phosphate-buffered saline (PBS, pH 7.4) was used throughout all washing and resuspension steps. It was prepared with sodium phosphate and sodium chloride, alongside optional inclusion of potassium phosphate and potassium chloride.

Density Gradient Medium:

Ficoll-Paque PLUS (GE Healthcare, Chicago, IL, USA), with a density of 1.077 g/mL, was used for isolating peripheral blood mononuclear cells (PBMCs) via density gradient centrifugation.

Fixative:

Cells were fixed in 4% paraformaldehyde (PFA; Sigma-Aldrich, St. Louis, MO, USA) in PBS for 10 min at room temperature to preserve cellular morphology and halt enzymatic degradation.

Fluorescent Labels:

Since we are still in the process of collecting sufficient cell data, fluorescence staining is currently used as an auxiliary labeling method. In the future, once the model becomes more stable, we plan to transition to a fully label-free approach.

EpCAM-FITC (BD Biosciences, San Jose, CA, USA): This was used to positively identify epithelial-derived CTCs.

CD45-PE (BioLegend, San Diego, CA, USA): This was used as a negative marker to identify and exclude leukocytes.

Hoechst 33342 (Thermo Fisher, Waltham, MA, USA): This is a nuclear dye that binds DNA and fluoresces blue under 350 nm excitation.

CTC identification was based on criteria adapted from the CellSearch^®^ system [40], whereby cells expressing EpCAM and Hoechst positivity but lacking CD45 expression were classified as CTCs, improving specificity by excluding hematopoietic cells.

#### 2.1.2. Sample Processing Procedure

Approximately 3 mL of Ficoll was pipetted below the filter insert in a Leucosep^®^ separation tube (Greiner Bio-One, Kremsmünster, Austria), followed by the careful layering of 2 mL of patient-derived whole blood along the tube wall. Samples were centrifuged at 800× *g* for 15 min to isolate the mononuclear cell layer, which includes lymphocytes, CTCs, and platelets. This intermediate layer was extracted and subjected to an additional centrifugation at 400× *g* for 10 min.

The resulting supernatant was reduced to 1.9 mL, followed by a PBS wash and centrifugation at 400× *g* for 6 min. The final cell pellet was resuspended in 100 μL of PBS and mixed with 200 μL of 4% PFA for a 10 min fixation. After washing with 700 μL of PBS, cells were sequentially stained in the dark with the following:

In total, 10 μL EpCAM-FITC, 5 μL CD45-PE (30–60 min), and 2.5 μL Hoechst 33342 (10 min) were used.

Post-staining, cells were washed with 900 μL PBS and resuspended. A 10 μL aliquot was used for cell counting, and the remaining sample was adjusted to a final concentration of $2\times 10^{4}$ cells/μL. The suspension was then loaded onto the in-house developed Self-Assembly Cell Array (SACA) chip for standardized imaging

Utilizing gravity and side capillary fluid forces within a 5 μm gap, the SACA chip organizes the cells into a flattened monolayer on its hydrophilic, Si$O_{2}$-coated polycarbonate substrate. This precise spatial configuration prevents cell overlapping and ensures a consistent focal plane across the entire scanning area. This arrangement allows each hyperspectral pixel to accurately reflect the biochemical signature of individual cellular components without the interference of spectral mixing from clustered cells.

**Figure 2:**
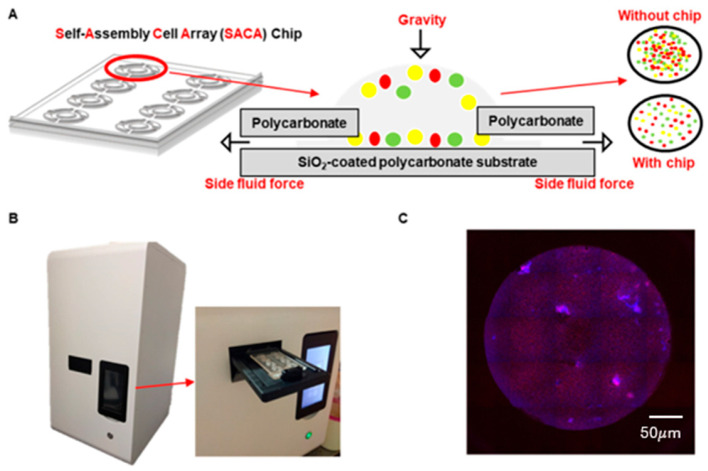
Schematic images of the SACA chip, automatic scanning platform, and output image. (**A**) Schematic images show the self-assembled cell array (SACA) chip applied in this study and the related principle. The hydrophilic bottom of the chip is made up of a SiO_2_-coated polycarbonate (PC) substrate. The input cells in solution spread on the bottom of the chip due to gravity and are pulled to keep a flattened monolayer by the side capillary fluid force due to the designed 5 μm gap and an outlet on each side, while other contents in solution are discharged by the side fluid force. (**B**) Images show the high-resolution automatic scanning platform (customized to fit the SACA chip) (CytoSCM imaging@cellenvision) applied in this study to detect and analyze CTC/CTM. (**C**) A stitched image of a well of the SACA chip with monolayered cells; the tile is scanned by the platform with all channels merged.

Samples deposited on the SACA chip were first scanned using an in-house customized automatic platform (CytoSCM imaging@cellenvision). This platform performed high-speed fluorescence scanning to identify and record the spatial coordinates of candidate cells. Subsequently, the chip was transferred to a dedicated hyperspectral imaging system, where the pre-recorded coordinates were used to capture label-free hyperspectral data cubes across 150 spectral bands. This coordinate-based relocation ensures precise spatial registration between the fluorescence ground truth and the hyperspectral data. Schematic images of the chip, scanning platform and output image were presented in Figure 2.

**Figure 3:**
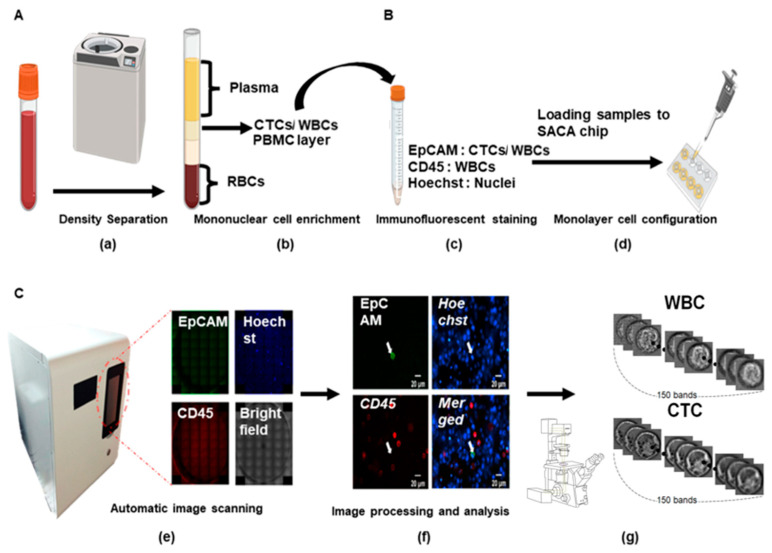
Schematic flowchart of the integrated SACA chip and label-free hyperspectral imaging (HSI) pipeline for CTC detection and classification. (**A**) Pre-processing stage including (**a**) density separation and (**b**) mononuclear cell enrichment. (**B**) Labeling and loading stage including (**c**) immunofluorescent staining with EpCAM, CD45, and Hoechst, and (**d**) Formation of a cell monolayer following the loading of the CTC/WBC-containing solution onto the SACA chip. (**C**). CTC detection: (**e**) Automated fluorescence scanning of the SACA chip using the CytoSCM platform, followed by image processing to identify target cells and record their precise coordinates. (**f**) Relocation of target cells by transferring the chip to the standalone hyperspectral system and navigating to the pre-recorded coordinates. (**g**) Acquisition of hyperspectral data cubes at the identified locations, followed by label-free spectral analysis and classification.

The complete workflow—comprising cell isolation, fixation, labeling, and scanning—is illustrated in Figure 3. The same protocol was strictly applied to both circulating tumor cells (CTCs) and white blood cells (WBCs) to ensure data consistency.

### 2.2. Automated Multi-Modal Imaging Platform

The SACA-chip-deposited sample was analyzed using a two-stage imaging workflow involving an in-house customized automatic scanning platform (CytoSCM imaging@cellenvision) and a standalone hyperspectral imaging (HSI) system. The scanning platform integrates high-resolution microscopic optics (IX71; Olympus, Tokyo, Japan) for initial cell localization.

In this workflow, the platform first automatically scans the SACA chip in fluorescence mode to identify the precise spatial coordinates of candidate CTCs based on EpCAM, CD45, and Hoechst markers. Following identification, the chip is transferred to a visible and near-infrared (VNIR) HSI system (SNAPSCAN VNIR B150U, imec, Leuven, Belgium). By utilizing the pre-recorded coordinates, the HSI system captures label-free hyperspectral cubes across 150 spectral bands (470–900 nm) at the exact same locations. This coordinate-based relocation process ensures reliable spatial registration between the fluorescence ground truth and the label-free hyperspectral data.

#### 2.2.1. Hyperspectral Camera Specifications

The SNAPSCAN VNIR B150U camera captures 150 spectral bands spanning 470–900 nm at 2.886 nm intervals. Operating in a line-scan mode, the system includes physically separated VNIR sensors to minimize crosstalk. Spectral acquisition is performed using a combination of a liquid crystal tunable filter (LCTF) and an acousto-optic tunable filter (AOTF), enabling precise frame-by-frame spectral capture.

#### 2.2.2. Hardware and Software Environment

All deep learning models and data processing tasks were implemented on a laptop workstation. The hardware configuration included an Intel Core i7-12700H CPU, 16 GB of DDR4 RAM, and an NVIDIA GeForce RTX 3060 Laptop GPU with 6 GB of VRAM. The software environment was established on Windows 11 using Python 3.12.2. Deep learning frameworks, specifically PyTorch 2.5.1, were utilized with NVIDIA CUDA 12.4 acceleration to facilitate the high-dimensional computation required for hyperspectral image classification.

#### 2.2.3. Camera Configuration and Calibration

White and Black References:

The system automatically captures a black reference. A manual white reference was acquired using a blank slide with PBS to ensure consistency under experimental imaging conditions. These references were used to correct for sensor noise and ambient light variability.

TDI Pixel Step:

Time Delay Integration (TDI) was set to 1 to reduce image distortion, improving spatial resolution at the expense of longer acquisition times.

Illumination Source:

A halogen lamp without an NIR filter was used to achieve full-spectrum VNIR imaging. Some fluctuations in intensity and color temperature were observed, especially in the blue region.

Optics:

Olympus UPLXAPO objectives were selected to minimize chromatic aberration and ensure image clarity across the full spectral range.

### 2.3. Deep Learning Models

To classify CTCs from hyperspectral data, two deep learning models were implemented: a pixel-level model focused on spectral features and a cell-level model that incorporated morphological characteristics. Both models were built upon ResNet architectures.

#### 2.3.1. A^2^S^2^K-ResNet

The A^2^S^2^K-ResNet model, short for Attention-Based Adaptive Spectral–Spatial Kernel ResNet, is a specialized deep learning architecture designed to address the challenges of high-dimensional, small-sample hyperspectral image classification [41]. In this study, we selected A^2^S^2^K-ResNet due to its ability to simultaneously leverage both spectral and spatial information, which is critical for distinguishing morphologically similar cell types such as circulating tumor cells (CTCs) and white blood cells (WBCs).

The model is built upon a ResNet backbone, incorporating several key components:

1. Adaptive Spectral–Spatial Kernels (A^2^S^2^K): These kernels are designed to automatically adjust their size and receptive field shape according to local spectral–spatial patterns. This adaptability enhances the model’s sensitivity to local heterogeneity, allowing it to better capture subtle variations in hyperspectral data, such as those found between CTCs and WBCs.
2. Attention Mechanisms: Both channel attention and spatial attention modules are integrated to dynamically weigh the importance of different spectral channels and spatial regions. This ensures that the model emphasizes biologically relevant features and suppresses redundant or noisy information, thereby improving classification robustness.
3. Multi-Scale Fusion Blocks: By extracting and combining features at multiple spatial and spectral resolutions, the model can capture both global context and fine-grained local differences—an essential capability for resolving subtle differences in cellular morphology and composition.

In our implementation, we adopted the full A^2^S^2^K-ResNet structure, including its spectral–spatial residual blocks, multi-scale fusion, and dual attention modules. This comprehensive design enables the model to effectively learn from limited hyperspectral data while maintaining high classification accuracy. The structure of the model is detailed in Supplementary Figure S3.

Overall, the A^2^S^2^K-ResNet was chosen for its superior capability in extracting discriminative features from complex hyperspectral images, making it well-suited for label-free classification of rare and heterogeneous cell populations in biomedical applications.

#### 2.3.2. Modified ResNet50

A modified ResNet50 model, pre-trained on ImageNet and subsequently fine-tuned on our dataset, was employed for structural image analysis [26]. The architecture comprises an initial 7 × 7 convolutional layer, followed by a max pooling layer, enabling early spatial feature extraction. This is followed by a series of residual blocks that facilitate deep hierarchical representation learning. The final stage includes a fully connected layer that reduces the feature dimensionality to 512, accompanied by a dropout layer with a rate of 0.6 to prevent overfitting. The model concludes with a SoftMax output layer configured for binary classification.

### 2.4. Model Evaluation Metrics

To comprehensively evaluate model performance, several key metrics were applied, including the confusion matrix, accuracy, precision, recall, average accuracy (AA), overall accuracy (OA), and the kappa statistic. The **confusion matrix** provides a detailed comparison of predicted versus actual classifications, indicating the counts of true positives (TP), true negatives (TN), false positives (FP), and false negatives (FN).

Accuracy measures the proportion of correctly classified instances over the total number of predictions.

$$Accuracy=\frac{TP+TN}{TP+FP+FN+TN}$$

Precision, or positive predictive value, quantifies the proportion of correctly predicted positive cases among all predicted positives.

$$Precision=\frac{TP}{TP+FP}$$

Recall, also known as sensitivity, assesses the model’s ability to identify actual positive cases.

$$Recall=\frac{TP}{TP+FN}$$

Average Accuracy (AA) represents the meaning of class-wise accuracies and is particularly useful for evaluating performance on imbalanced datasets, while **Overall Accuracy (**OA**)** reflects the total proportion of correct predictions across all classes. Lastly, the **kappa statistic** measures the level of agreement between predicted and actual classifications beyond chance. It is calculated as follows:

$$kappa=\frac{p_{o}-p_{e}}{1-p_{e}}\;\;\;p_{o}=\frac{TP+TN}{TP+FP+FN+TN}\;\;$$

where

$$\;\;p_{e}=\frac{\left(TP+FN\right)\left(TP+FP\right)+\left(TN+FP\right)\left(TN+FN\right)}{{\left(TP+FP+FN+TN\right)}^{2}}$$

Together, these metrics provide a robust and multidimensional evaluation of the classification model’s performance.

## 3. Results

This section presents the outcomes of classification experiments aimed at distinguishing circulating tumor cells (CTCs) from white blood cells (WBCs) using both spectral and structural features. Quantitative metrics and visualizations are provided to assess model performance under various conditions.

### 3.1. Classification Based on Spectral Information

We first explored the use of hyperspectral information to distinguish CTCs from WBCs, based on their subtle spectral differences. The A^2^S^2^K-ResNet model was initially employed for this task and demonstrated strong performance in differentiating cultured cancer cell lines from WBCs. The model effectively captured distinct spectral features that enabled accurate classification under controlled conditions. However, when applied to patient-derived CTCs, classification performance declined, suggesting that spectral differences between CTCs and WBCs were less pronounced in real biological samples.

#### 3.1.1. Classification of Cell Lines vs. WBCs

**Figure 4:**
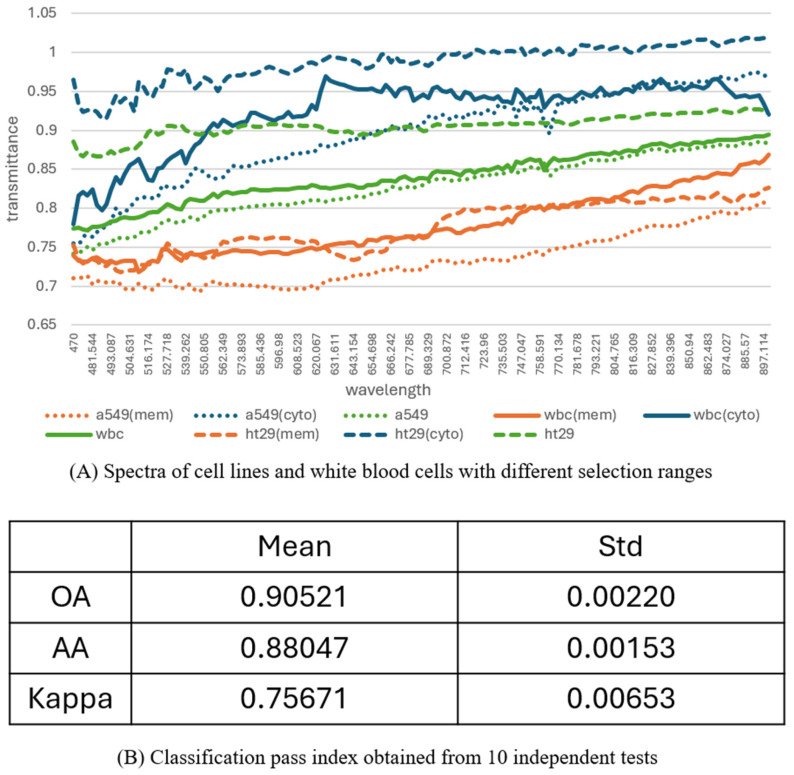
Spectral analysis and classification results of cell lines and WBCs. Spectral features of cytoplasm and whole cells are visualized alongside classification performance metrics.

As shown in Figure 4A, spectral curves were extracted from both whole-cell regions and cytoplasmic areas. Specifically, the blue curve represents the cytoplasm of WBCs, the orange-red curve corresponds to the cytoplasm of HT29 cells, the green curve indicates the full WBC, and the yellow curve represents the full HT29 cell. We observed that averaging spectra over the entire cell area smoothed out intercellular differences, reducing discriminatory power. In contrast, spectral curves extracted specifically from the cytoplasm or internal organelles revealed greater variation between cell types and preserved distinguishing features.

This phenomenon can be explained by the composition of cellular components. While cell membranes are primarily composed of phospholipids and proteins—largely consisting of carbon, hydrogen, nitrogen, and oxygen—the spectral differences among them are relatively minor due to their similar molecular makeup. In contrast, the cytoplasm contains a more diverse array of biomolecules, making it a richer source of discriminative spectral signals. With adequate sensitivity, hyperspectral systems can even detect spectral signatures of molecular bonds. Therefore, focusing on cytoplasmic regions enhances the ability to differentiate between cell types. Additional spectral comparisons between WBCs and HT29 cells are provided in Supplementary Figure S4, further highlighting the distinct features present in the cytoplasm.

Figure 4B presents quantitative classification results, including overall accuracy (OA), average accuracy (AA), and Cohen’s kappa values, derived from 10 independent test runs. These metrics were computed on a pixel-wise basis, meaning each pixel was individually classified by the model. The results demonstrate that the model achieved an AA exceeding 80%, successfully distinguishing HT29 cells from WBCs with high precision. The accompanying kappa values further validate the model’s reliability by accounting for agreement beyond random chance.

**Figure 5:**
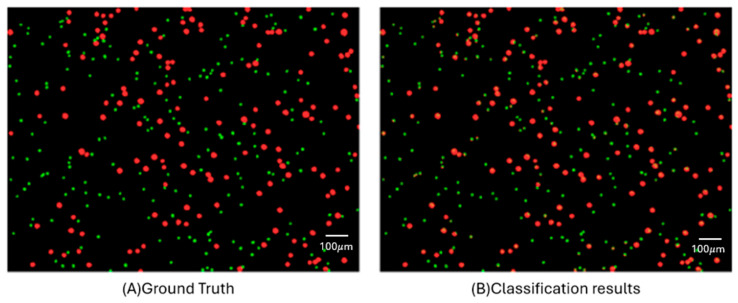
Pixel-Wise Comparison of Labeling and Prediction: visual comparison between (**A**) and (**B**). Red dots represent cancer cells (e.g., HT29), and green dots represent white blood cells (WBCs). The model output closely matches the ground truth, demonstrating strong classification performance at the pixel level.

Figure 5 shows a visual representation of pixel-level classification outcomes. In this image, red pixels denote regions identified as cancer cells (HT29/A549), while green pixels indicate areas classified as WBCs. This color-coded prediction map clearly illustrates the model’s ability to delineate the boundaries between different cell types, demonstrating its practical potential for hyperspectral-based cell classification.

#### 3.1.2. Classification of CTCs vs. WBCs

**Figure 6:**
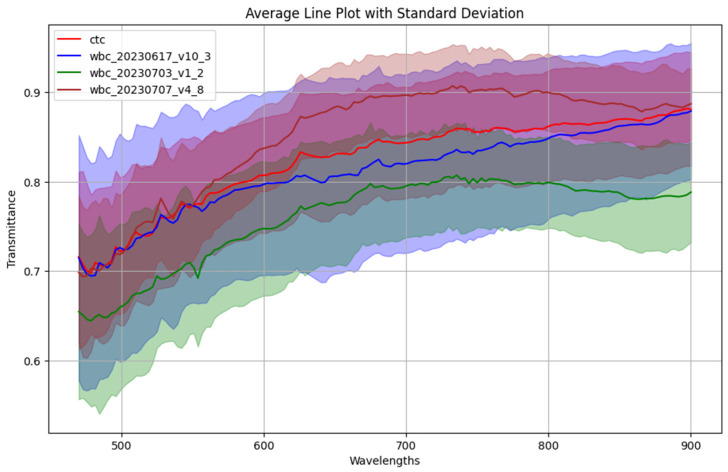
Classification of Circulating Tumor Cells (CTCs) and White Blood Cells (WBCs) Based on Cytoplasmic Spectra and Partial Band Analysis. This figure illustrates the spectral variability between CTCs and WBCs using cytoplasmic region data and evaluates classification outcomes across selected spectral subranges. The left panel highlights the overlap and variability in spectral profiles, particularly within WBC samples.

To enhance discriminatory power, spectral data were extracted specifically from the cytoplasmic region at the center of each cell, where meaningful biochemical variation is most likely to occur. However, unlike the cell line experiments, this approach did not yield clearly distinguishable spectral patterns between circulating tumor cells (CTCs) and white blood cells (WBCs). As illustrated in Figure 6, closer inspection of the WBC spectra revealed considerable intra-class variability across different batches of samples. In many cases, the spectral variation within WBCs exceeded the subtle differences observed between WBCs and CTCs. This high degree of variability makes it challenging to train a reliable classification model, particularly when inter-class differences are less pronounced than intra-class fluctuations.

To address this issue, we explored the effectiveness of using partial spectral band subsets. The hyperspectral imaging system used in our experiments captures data across 150 spectral bands from 470 nm to 900 nm. Since cells exhibit low absorbance in the visible spectrum—appearing largely transparent under standard imaging conditions—we divided the full spectral range into three equal segments: short, medium, and long wavelengths (each containing 50 bands). This division served as a form of dimensionality reduction and allowed for targeted analysis of potentially more informative spectral regions.

For these tests, a dataset of 30 CTCs was augmented through weighting to approximate the number of WBCs (~300) typically present in a single hyperspectral frame. While the overall classification accuracy improved by less than 3% compared to using all 150 bands, this marginal gain reflects the inherent limitations of working with a small number of positive (CTC) samples. In this context, recall—defined as the ratio of true positives to the sum of true positives and false negatives (TP/[TP + FN])—is a more appropriate metric for evaluating model performance on rare classes.

In the short-wavelength region, the model achieved a recall of approximately 40%, indicating that around half of the predicted pixels as CTCs were correctly classified.

**Figure 7:**
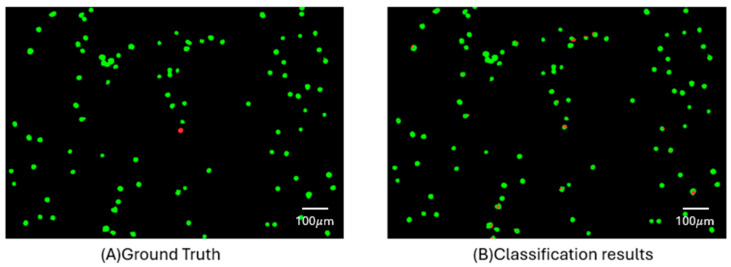
The panel presents pixel-level prediction maps generated from the short-wavelength bands (470–611.4 nm). Subfigure (**A**) Ground truth (red: CTCs; green: WBCs); (**B**) Classification results. Most of the annotated CTCs are correctly identified, demonstrating the model’s improved recall under this spectral range. Notably, the number of false positives remains low, highlighting the model’s robustness in distinguishing rare CTCs from abundant WBCs despite their spectral similarity. These findings validate the effectiveness of the short-band strategy in enhancing detection accuracy while minimizing misclassification in label-free scenarios.

As illustrated in Figure 7, pixel-level prediction maps derived from the short-wavelength range demonstrate the model’s improved ability to identify CTCs. The left panel shows the ground truth annotations, while the right panel presents the corresponding classification results. Despite significant spectral overlap with WBCs, the model successfully identified many CTCs with minimal false positives. This visual evidence supports quantitative recall metrics and highlights the partial-band approach’s practical potential for enhancing detection in label-free settings. Although modest, this performance represents a substantial improvement over earlier trials, where recall rates ranged from 0% to 10%. Moreover, as shown in Table 1, the rate of WBC pixels misclassified as CTCs remained relatively low, further supporting the model’s practical potential. A detailed breakdown of accuracy and recall across all individual wavelengths is provided in Supplementary Figure S1, which highlights the most informative spectral bands and illustrates the variability of model performance across the full spectrum.

### 3.2. Classification Based on Cellular Structures

To complement spectral analysis, we explored the use of structural image features for classifying circulating tumor cells (CTCs) and white blood cells (WBCs). By applying supervised learning techniques, we aimed to enable the model to detect subtle morphological differences between cell types—features that are often imperceptible through traditional image analysis methods. This approach facilitates more precise and robust cell classification.

#### 3.2.1. Classification of CTCs and WBCs Using Single Wavelength

**Figure 8:**
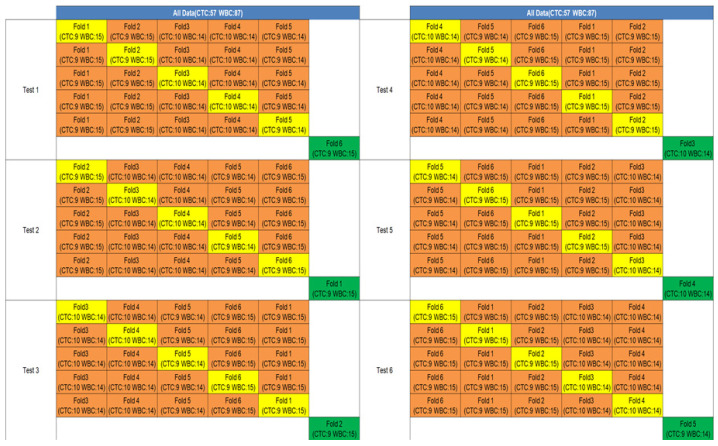
Classification of Circulating Tumor Cells (CTCs) and White Blood Cells (WBCs) Using Single-Band Images. This figure presents the classification performance of a deep learning model trained on grayscale images derived from a single spectral band. The results demonstrate that even with limited spectral input, structural features extracted from individual wavelengths are sufficient to enable effective differentiation between CTCs and WBCs. Consistent accuracy, recall, and precision across multiple trials confirm the feasibility and robustness of single-band structural analysis.

Figure 8 presents the classification results obtained using single-band grayscale images. Each image corresponds to a single wavelength channel from the hyperspectral cube, and the model leverages morphological features captured at that wavelength to differentiate between CTCs and WBCs.

The dataset consisted of 56 CTCs and 87 WBCs. To assess generalizability, a subset of 5 CTCs and 5 WBCs was randomly selected as a held-out test set, while the remaining samples were used for model training. A five-fold cross-validation strategy was employed to tune model parameters and evaluate stability. Following training, the best-performing model configuration was applied to the test set.

In the initial trial, the model achieved an accuracy of 80%, a perfect recall of 100%, and a precision of 71.4%, indicating that all CTCs were correctly identified, albeit with some false positives. Across six independent trials, the model consistently maintained strong performance, with accuracy, recall, and precision all exceeding 80%. These results confirm the effectiveness and robustness of single-band structural analysis in distinguishing CTCs from WBCs using deep learning.

The quantitative results of the six independent validation trials are presented in Table 2.

This table summarizes model performance for each cross-validation fold, including accuracy, recall, and precision. The results demonstrate strong and consistent classification performance, with all metrics averaging above 87%. These findings confirm the reliability of single-band structural analysis for distinguishing circulating tumor cells (CTCs) from white blood cells (WBCs).

#### 3.2.2. Classification of CTCs and WBCs Using Multiple Bands

**Figure 9:**
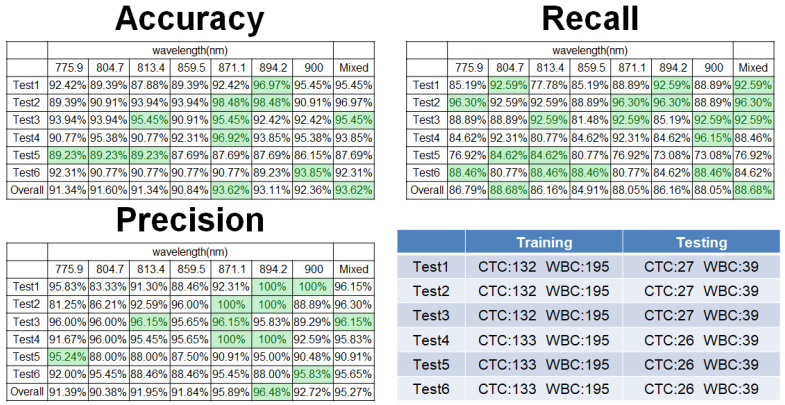
Classification of Circulating Tumor Cells (CTCs) and White Blood Cells (WBCs) Using a Hybrid Model Based on Multiple Wavelengths. This figure shows the classification performance of a hybrid model that integrates predictions from seven individually optimized spectral bands. Each wavelength-specific model performs independent classification, and the final label is determined through majority voting. This ensemble approach improves overall robustness, reduces the influence of noisy or suboptimal bands, and enhances prediction reliability across varied cellular samples.

Figure 9 illustrates the performance of a hybrid classification model that integrates structural information from seven carefully selected spectral bands. Each band independently contributes to classification through a dedicated model, and the final decision for each input cell is determined by a majority voting strategy, enhancing robustness and reliability.

To identify the most informative wavelengths, we first evaluated the classification performance of individual bands across the 470–900 nm spectral range. Using fixed training and test datasets, we conducted three independent experiments to assess band-specific recognition accuracy. The seven wavelengths yielding the highest individual accuracies were selected for inclusion in the hybrid model. The voting mechanism used to combine predictions from these seven channels is schematically illustrated in Supplementary Figure S2.

This ensemble approach improved model generalization while reducing computational complexity compared to full-spectrum analysis. For this experiment, we used a dataset comprising 159 circulating tumor cells (CTCs) and 234 white blood cells (WBCs). The hybrid model demonstrated strong classification performance by leveraging complementary structural features across multiple bands. Compared to the average accuracy of 88.2% obtained using single-band analysis, the multi-band ensemble approach improved the classification accuracy to over 93.5%, with a precision exceeding 92%. This represents an improvement of more than 5% in accuracy, validating the effectiveness of the structural fusion and majority voting strategy. These findings confirm the significant advantage of multi-band integration over single-band approaches in achieving robust and reliable classification of CTCs and WBCs.

## 4. Discussion

The experimental results indicate that although the hybrid model did not achieve the highest recognition score in every individual trial, it consistently outperformed single-band approaches in overall classification performance. This consistency underscores the model’s robustness and affirms its alignment with the study’s original objectives of enhancing stability and reliability in CTC detection.

A major advantage of the hybrid model is its ability to integrate spectral information across multiple informative wavelengths. While single-band models may achieve high performance under ideal conditions, they are often limited by poor generalizability and increased susceptibility to noise or variability within individual spectral channels. By contrast, the majority voting mechanism employed in the hybrid model mitigates these limitations by fusing the predictions of multiple wavelength-specific classifiers. This ensemble approach not only compensates for the weaknesses of individual bands but also enhances the model’s resilience to sample heterogeneity and spectral anomalies.

Furthermore, the multi-wavelength strategy provides a more comprehensive and information-rich representation of cellular features. The complementary nature of spectral data across different wavelengths enables the model to capture nuanced differences in both cell morphology and biochemical composition. This is particularly valuable when classifying rare and heterogeneous populations such as CTCs, where minor spectral distinctions can be easily masked by biological noise or intra-class variation. By leveraging spectral diversity, the hybrid model significantly improves recognition reliability under variable experimental conditions.

Furthermore, the integration of the SACA system (CytoSCM imaging@cellenvision) provides a fundamental technical advantage over conventional label-free HSI methods that rely on simple cell smears or droplet-based slide preparation. In traditional preparations, cell clustering and overlapping often lead to significant spectral mixing, where the captured signal is a composite of multiple cells or debris, thereby reducing the discriminative power of deep learning models. By contrast, the SACA chip’s 5 μm gap design utilizes fluidic and gravitational forces to sequester cells into a precisely organized, flattened monolayer. This hardware-level spatial control ensures that each hyperspectral pixel corresponds to a single cell layer, effectively minimizing biological noise and enabling the A2S2K-ResNet model to extract high-purity cytoplasmic features. Furthermore, unlike static slide imaging, the SACA platform’s automated scanning and coordinates-based registration allow for the potential recovery of identified CTCs via its integrated micro-pump system for subsequent functional analysis, a capability that significantly enhances the clinical utility of our label-free detection pipeline.

Nevertheless, the effectiveness of the hybrid approach depends critically on the careful selection of spectral bands. Including non-informative or noisy wavelengths in the ensemble may dilute classification accuracy and introduce prediction errors. As such, band selection should be guided by empirical performance metrics and targeted feature analysis to ensure that only the most discriminative wavelengths are incorporated into the model.

Another important consideration is the impact of class imbalance on model performance. In this study, the significantly lower prevalence of CTCs compared to WBCs in the dataset led to reduced recall for the minority class. Supplementary Figure S6 illustrates how different CTC/WBC ratios influence accuracy, recall, and precision. These findings highlight the importance of addressing class imbalance—either through balanced sampling, synthetic data augmentation, or by applying weighted loss functions—to improve sensitivity without compromising overall precision.

In summary, this study demonstrates that combining spectral and structural features with a carefully optimized hybrid classification framework leads to significant improvements in the detection of circulating tumor cells. The integration of multi-band spectral data, attention to band selection, and mitigation of class imbalance contribute to a model that is not only accurate but also stable and adaptable. These advancements position the proposed system as a promising tool for future clinical applications in non-invasive liquid biopsy and cancer diagnostics.

## 5. Conclusions

In this study, we developed a machine learning-based framework to accurately identify and classify circulating tumor cells (CTCs) and white blood cells (WBCs) from hyperspectral images of cancer patient blood samples. Given the clinical relevance of CTCs in cancer metastasis and recurrence, this work has significant implications for early diagnosis and treatment planning.

Initially, we investigated pixel-level spectral features for cell-type discrimination. Although this approach achieved promising results when classifying cancer cell lines versus WBCs, its performance on patient-derived CTCs was limited, likely due to subtle spectral differences between CTCs and WBCs. To address this, we employed a modified ResNet50 deep learning model to extract morphological features from single-wavelength images, demonstrating that structural characteristics could provide complementary discriminatory power.

Building upon this, we introduced a hybrid model that integrates classification results from multiple informative wavelengths using an ensemble learning strategy. This approach not only improved overall classification accuracy but also enhanced performance stability across experiments. By aggregating spectral–spatial features, the model mitigates the limitations of single-band analysis and enables a more comprehensive representation of cellular characteristics.

Although the current framework offers a viable path toward label-free CTC detection, certain challenges still need to be addressed. These include the small number of patient-derived CTC samples, spectral variability within WBC populations, and the continued need for manual annotation during model training. Nonetheless, our results highlight the potential of hyperspectral imaging combined with deep learning for future applications in clinical liquid biopsy and real-time cancer monitoring.

## Figures and Tables

**Table 1:** Interval band accuracy and recall.

Test Data	Wavelength	The Number of CTCs	Weight	Accuracy	CTC Recall
A	470–611.4	30	10	52.4%	46.7%
	624.3–755.7	30	10	51.0%	6.5%
	758.6–900	30	10	49.9%	0.0%
B	470–613	30	10	52.9%	34.0%
	614.3–755.7	30	10	54.6%	21.6%
	758.6–900	30	10	49.8%	0.0%

**Table 2:** Accuracy, Recall, and Precision Across Six Independent Trials Using Single-Band Image Classification.

Test	Accuracy	Recall	Precision
1	87.50%	87.86%	87.06%
2	66.67%	65.71%	65.71%
3	87.50%	86.43%	87.78%
4	95.83%	96.67%	95.00%
5	91.67%	91.11%	91.11%
6	100.00%	100.00%	100.00%
Average	88.195%	87.963%	87.777%

## Data Availability

The datasets generated and/or analyzed during the current study are available from the corresponding author on reasonable request.

## Supplementary Materials

The following supporting information can be downloaded at https://www.mdpi.com/article/10.3390/mi17040472/s1. Figure S1: Identification of the most informative spectral bands across the full spectrum; Figure S2: Schematic diagram of the majority voting mechanism used in the multi-band ensemble model; Figure S3: Detailed architecture of the A^2^S^2^K-ResNet model including spectral-spatial residual blocks and dual attention modules; Figure S4: Spectral comparison between white blood cells (WBCs) and HT29 cell lines in the cytoplasmic region; Figure S5. Classification Performance Using Suboptimal Wavelengths; Figure S6: Impact of varying CTC/WBC ratios on model accuracy, recall, and precision; Figure S7. Grayscale Images of CTCs and WBCs at a Single Wavelength; Figure S8: Collaborative workflow for patient-derived sample collection and EMT analysis.
