# Supplementary material for: Automated Label-Free Classification of Circulating Tumor Cells and White Blood Cells Using Hyperspectral Imaging and Deep Learning on Microfluidic SACA Chip System
Source: Micromachines (Basel). 2026 Apr 14;17(4):472. doi: 10.3390/mi17040472 (PMC13118043; doi:10.3390/mi17040472)
Supplement: Supplementary file 1 [file micromachines-17-00472-s001.zip › micromachines-4235994-supplementary.pdf]

Automated Label-Free Classification of Circulating Tumor Cells and White Blood Cells Using Hyperspectral Imaging and Deep Learning

Shun-Chi Wu[1], Jon-Nan Chiu[1], Yi-Wen chen[1], [2]Ou-Yang Mang, and *Fan-Gang Tseng[1,3]

[1] *Department of Engineering and System Science, National Tsing Hua University, Hsinchu, Taiwan, R.O.C.*

[2]*Department of Electronics and Electrical Engineering, National Yang Ming Chiao Tung University, Hsinchu, Taiwan, R.O.C.*

[3]*Research Center for Applied Sciences, Academia Sinica, Taipei, Taiwan R.O.C.*

**Corresponding: fangang@ess.nthu.edu.tw*

Supplementary

Figure S1. presents the classification accuracy and recall for each spectral band within the 470 – 900 nm range. Peaks in the curves represent wavelengths with high discriminative capability for distinguishing circulating tumor cells (CTCs) from white blood cells (WBCs), while troughs indicate bands with lower reliability or potential noise interference. These results informed the selection of the seven most informative wavelengths used in the hybrid classification model.

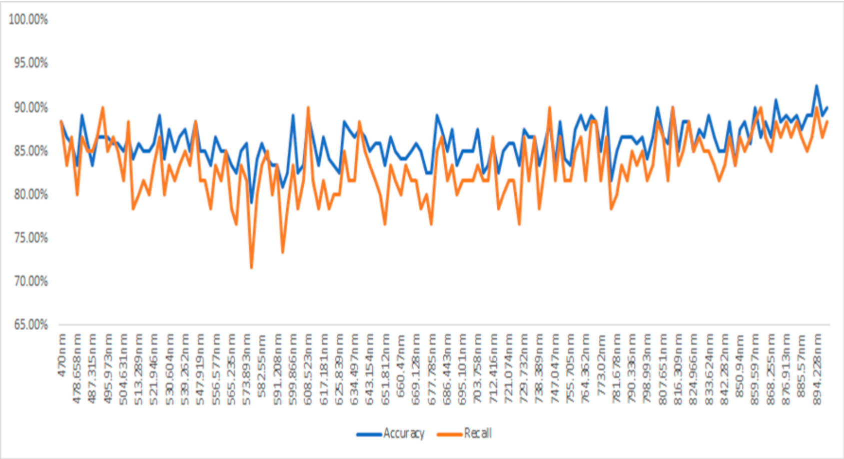

	Accuracy	Recall
894.228nm	92.50%	90.00%
871.141nm	90.83%	88.33%
804.765nm	90.00%	88.33%
775.906nm	90.00%	86.67%
813.423nm	90.00%	90.00%
859.597nm	90.00%	88.33%
900nm	90.00%	88.33%
481.544nm	89.17%	86.67%
524.832nm	89.17%	86.67%
599.866nm	89.17%	83.33%
608.523nm	89.17%	90.00%
680.671nm	89.17%	85.00%
761.477nm	89.17%	86.67%
767.248nm	89.17%	88.33%
833.624nm	89.17%	85.00%
876.913nm	89.17%	88.33%
882.685nm	89.17%	88.33%
888.456nm	89.17%	85.00%
891.342nm	89.17%	86.67%
897.114nm	89.17%	86.67%

Figure S1. Accuracy and Recall Across Individual Wavelengths (470–900 nm).

Figure S2. illustrates the majority voting strategy employed in the hybrid classification model. Each of the seven selected wavelengths independently performs cell classification based on spectral and structural features. The final label for each input cell is determined by aggregating these individual predictions through majority voting. This ensemble approach enhances both the robustness and accuracy of CTC – WBC classification by mitigating the influence of noisy or underperforming spectral bands.

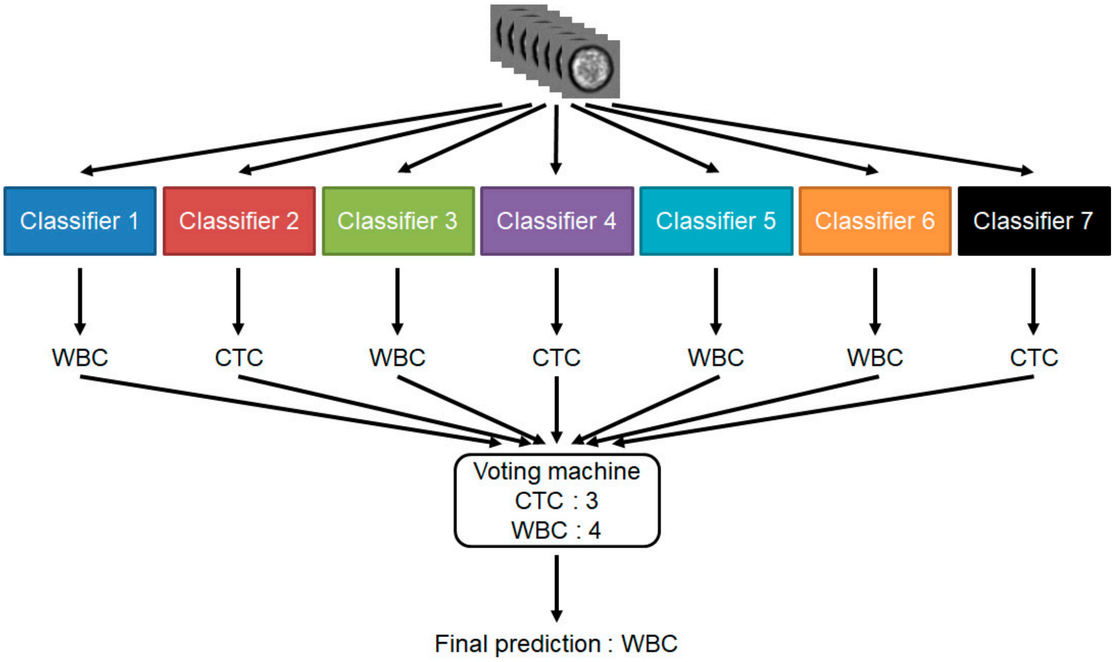

Figure S2. Schematic Diagram of the Hybrid Model Voting Mechanism.

Figure S3. illustrates the structure of the $A^2 S^2$ K-ResNet model, which integrates adaptive spectral – spatial kernels with attention mechanisms tailored for hyperspectral image analysis. The architecture includes multi-scale feature extraction blocks, channel attention, and spatial attention modules, enabling effective learning from high-dimensional spectral data. These components work in tandem to enhance the model’s ability to capture both spectral and spatial features critical for accurate classification of circulating tumor cells (CTCs) and white blood cells (WBCs).

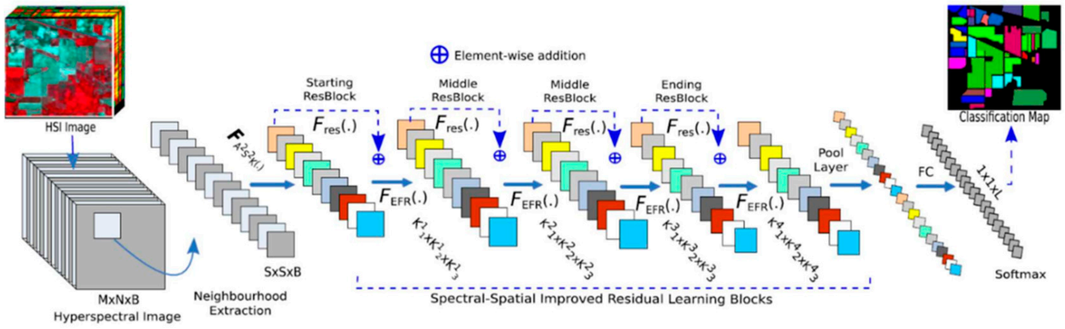

Figure S3. Architecture of the A2S2K-ResNet Model.

Figure S4. compares the reflectance spectra of WBCs and HT29 colorectal cancer cell lines, analyzing both whole-cell and cytoplasmic regions. The cytoplasmic spectra exhibit more pronounced differences between cell types, in contrast to the smoother and less distinctive patterns observed in whole-cell averages. These findings support the strategy of focusing on cytoplasmic regions to enhance spectral-based classification performance.

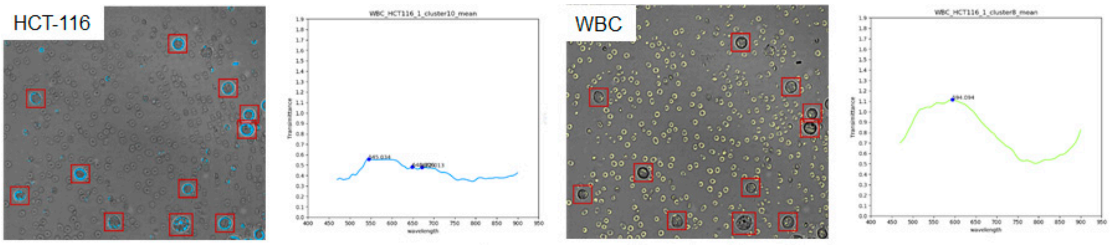

AP (TumorCell)	AP (WBC)	mAP	epoch
82.76	52.58	67.67	5
89.52	58.47	74.00	10
95.62	84.92	90.27	15
87.98	65.18	76.58	20
65.87	47.00	56.44	5
78.14	52.40	65.27	10
80.96	65.05	73.00	15
78.83	58.43	68.63	20
82.01	49.74	65.88	5
82.48	54.38	68.43	10
99.41	87.77	93.59	15
78.49	70.47	74.48	20
78.63	72.14	75.39	5
82.60	76.11	79.35	10
79.11	88.89	84.00	15
86.37	84.37	85.37	20

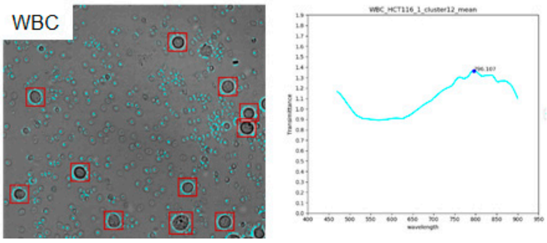

Figure S4. Spectral Comparison Between White Blood Cells (WBCs) and HT29 Cell Lines.

Figure S5. presents the classification results obtained by combining multiple low-performing spectral bands. The observed decrease in accuracy and increase in misclassification rates emphasize the critical role of wavelength selection in hyperspectral analysis. These results validate the band selection strategy adopted in this study, highlighting that inclusion of weak or noisy wavelengths can negatively impact model performance.

Accuracy

	wavelength(nm)							
	568.1	576.8	594.1	602.8	677.8	715.3	778.8	Mixed
Test1	89.39%	81.82%	83.33%	86.36%	92.42%	90.91%	80.30%	90.91%
Test2	90.91%	89.39%	90.91%	90.91%	90.91%	92.42%	92.42%	96.97%
Test3	86.36%	90.91%	87.88%	89.39%	95.45%	93.94%	93.94%	93.94%
Test4	87.69%	90.77%	90.77%	83.08%	89.23%	92.31%	90.77%	90.77%
Test5	84.62%	86.15%	89.23%	90.77%	87.69%	86.15%	89.23%	89.23%
Test6	93.85%	95.38%	96.92%	92.31%	95.38%	95.38%	90.77%	96.92%
Overall	88.80%	89.06%	89.82%	88.80%	91.86%	91.86%	89.57%	93.13%

Recall

	wavelength(nm)							
	568.1	576.8	594.1	602.8	677.8	715.3	778.8	Mixed
Test1	81.48%	70.37%	70.37%	77.78%	85.19%	77.78%	59.26%	81.48%
Test2	88.89%	85.19%	92.59%	88.89%	92.59%	92.59%	88.89%	96.30%
Test3	85.19%	88.89%	77.78%	81.48%	92.59%	85.19%	85.19%	92.59%
Test4	76.92%	84.62%	80.77%	61.54%	84.62%	80.77%	80.77%	80.77%
Test5	84.62%	84.62%	88.46%	88.46%	80.77%	76.92%	84.62%	84.62%
Test6	92.31%	88.46%	92.31%	88.46%	88.46%	88.46%	88.46%	92.31%
Overall	84.91%	83.65%	83.65%	81.13%	87.42%	83.65%	81.13%	88.05%

Precision

	wavelength(nm)							
	568.1	576.8	594.1	602.8	677.8	715.3	778.8	Mixed
Test1	91.67%	82.61%	86.36%	87.50%	95.83%	100%	88.89%	95.65%
Test2	88.89%	88.46%	86.21%	88.89%	86.21%	89.29%	92.31%	96.30%
Test3	82.14%	88.89%	91.30%	91.67%	96.15%	100%	100%	92.59%
Test4	90.91%	91.67%	95.45%	93.75%	88.00%	100%	80.77%	80.77%
Test5	78.57%	81.48%	85.19%	88.46%	87.50%	86.96%	88.00%	88.00%
Test6	92.31%	100%	100%	91.30%	100%	100%	88.46%	100%
Overall	87.10%	88.67%	90.48%	90.00%	92.05%	95.68%	89.58%	92.11%

	Training	Testing
Test1	CTC:132 WBC:195	CTC:27 WBC:39
Test2	CTC:132 WBC:195	CTC:27 WBC:39
Test3	CTC:132 WBC:195	CTC:27 WBC:39
Test4	CTC:133 WBC:195	CTC:26 WBC:39
Test5	CTC:133 WBC:195	CTC:26 WBC:39
Test6	CTC:133 WBC:195	CTC:26 WBC:39

Figure S5. Classification Performance Using Suboptimal Wavelengths.

Figure S6. illustrates how varying the ratio of circulating tumor cells (CTCs) to white blood cells (WBCs) in the training dataset affects key performance metrics, including classification accuracy, recall, and precision. The results show that increasing the relative representation of CTCs enhances sensitivity (recall) but may lead to a decrease in overall precision. These findings highlight the importance of addressing class imbalance to optimize model performance for rare cell detection.

	CTC40 + WBC80			CTC80 + WBC80			CTC80 + WBC160		
	Accuracy	Recall	Precision	Accuracy	Recall	Precision	Accuracy	Recall	Precision
Test1	87.5%	75%	100%	90%	90%	90%	92.5%	90%	94.74%
Test2	85%	75%	93.75%	87.5%	80%	94.12%	90%	80%	100%
Test3	92.5%	90%	94.74%	92.5%	95%	90.48%	92.5%	85%	100%
Average	**88.33%**	**80%**	**96%**	**90%**	**88.33%**	**91.38%**	**91.67%**	**85%**	**98.08%**

Figure S6. Impact of CTC/WBC Ratio on Model Performance.

Figure S7. displays representative grayscale images of circulating tumor cells (CTCs) and white blood cells (WBCs) captured at a single wavelength (e.g., 640 nm). The images highlight subtle differences in cell morphology and intensity patterns under monochromatic illumination. These variations provide valuable visual cues that can aid both deep learning－based model training and manual cell annotation.

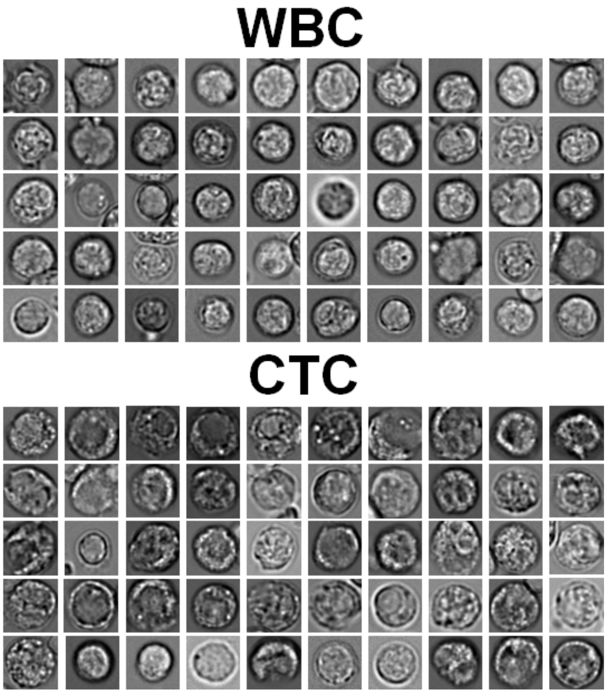

Figure S7. Grayscale Images of CTCs and WBCs at a Single Wavelength.

Figure S8. The samples collected for this study were collected from a clinical research project conducted in collaboration with Taipei Veterans General Hospital (IRB code: 2023-08-002CC), mainly for patients with stage III and IV colorectal cancer. As can be seen from Figure H (a), most patients are in stage III and IV, with male patients being the majority and a relatively low proportion of female patients. Figure H (b) shows the age and gender distribution of the patients, with the age range of the samples mainly falling between 41 and 80 years old, with the largest number of patients aged 61–70 years

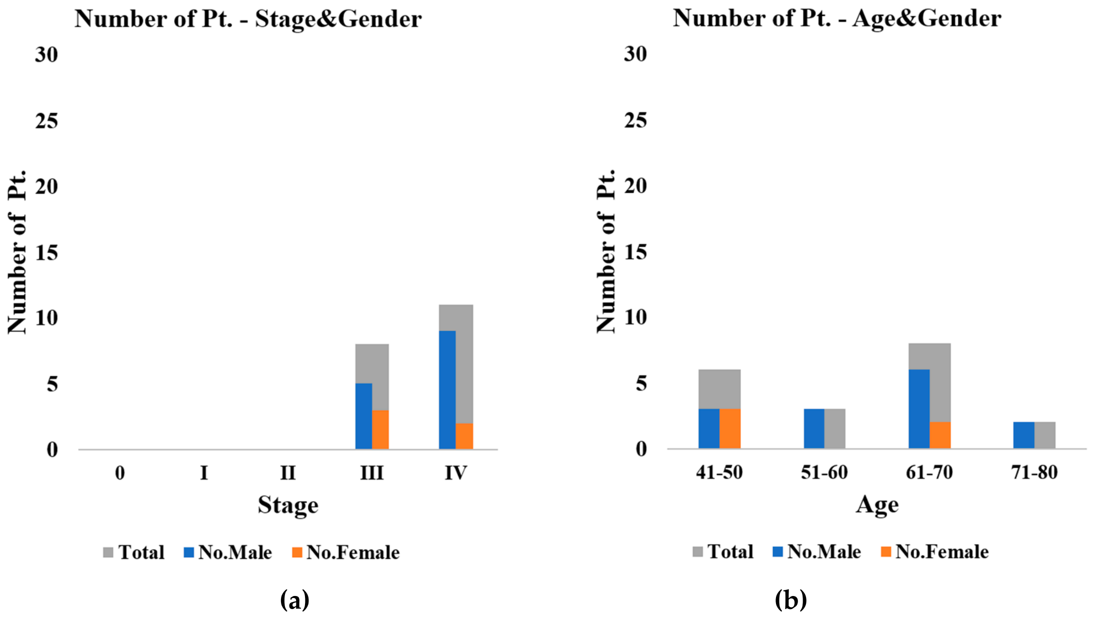

Figure S8. Clinical Stage and Age Distribution of Colorectal Cancer Patients
